# In Planta Detection of *Beauveria bassiana* (Ascomycota: Hypocreales) Strains as Endophytes in Bean (*Phaseolus vulgaris* L.)

**DOI:** 10.3390/plants13010022

**Published:** 2023-12-20

**Authors:** Teodora Cavazos-Vallejo, José Alberto Valadez-Lira, Alonso A. Orozco-Flores, Ricardo Gomez-Flores, María Julissa Ek-Ramos, Deyanira Quistián-Martínez, Juan Manuel Alcocer-González, Patricia Tamez-Guerra

**Affiliations:** 1 Departamento de Microbiología e Inmunología, Facultad de Ciencias Biológicas (FCB), Universidad Autónoma de Nuevo León (UANL), Avenida Pedro de Alba S/N, Ciudad Universitaria, San Nicolás de los Garza C.P. 66451, Nuevo León, Mexico; teodora.cavazosvl@uanl.edu.mx (T.C.-V.); jose.valadezlr@uanl.edu.mx (J.A.V.-L.); aorozcof@uanl.edu.mx (A.A.O.-F.); ricardo.gomezfl@uanl.edu.mx (R.G.-F.); maria.ekramos@uanl.edu.mx (M.J.E.-R.); juan.alcocergn@uanl.edu.mx (J.M.A.-G.); 2 Departamento de Botánica, Facultad de Ciencias Biológicas, Universidad Autónoma de Nuevo León, Avenida Pedro de Alba S/N, Ciudad Universitaria, San Nicolás de los Garza C.P. 66451, Nuevo León, Mexico; deyanira.quistianmrt@uanl.edu.mx

**Keywords:** entomopathogenic fungus, nested PCR, beans, molecular detection, group I introns

## Abstract

*Beauveria bassiana* (*B. bassiana*) is a significant entomopathogenic fungus (EPF) in agriculture as a sprayable biocontrol agent. It has the potential to be established as an endophyte (ENP) in various crops, resulting in beneficial effects for the host plants, including resistance to pest insects and increased growth and yield. However, it is not known whether a *B. bassiana* strain has such a favorable impact on the plant, since it is a common soil microorganism. Therefore, techniques that allow strain monitoring will be advantageous. To date, methods for detecting or monitoring a specific EPF strain after external application are scarce. In the present study, an in planta nested PCR technique was standardized to differentiate between three *B. bassiana* strains (GHA, PTG4, and BB37) established as endophytes in bean plants under laboratory conditions by detecting the insertion profile of four group I introns located in the 28S gene of *B. bassiana* ribosomal DNA. This technique recognized a distinct pattern of bands of different sizes for each strain, with a sensitivity of 1 pg per 10 ng of plant DNA. This molecular approach may be more effective monitoring *B. bassiana* strains after application to evaluate their significance on crops.

## 1. Introduction

Endophytes (ENP) are microorganisms (actinomycetes, bacteria, or fungi) that asymptomatically live inside plant tissues [1,2]. Some protect host plants from stress and promote their growth [3,4]. In addition to *Beauveria bassiana*’s potential as an entomopathogen, this fungus establishes as an endophyte, after being naturally implanted in plant tissues or artificially applied, which protects from herbivores [5,6,7], pathogens [8,9], or abiotic stress, among others [10,11,12]. 

Studies of the artificial application of *B. bassiana* have shown distinct activities and effects of strains on plants [13,14,15]. Therefore, it is necessary to standardize techniques to differentiate or detect strains to confirm that the positive effect obtained is due to the applied strain rather than a native one since this fungus is a microorganism commonly found in soil, even in semi-arid areas [16]. 

*Beauveria bassiana* has been artificially established in monocotyledonous [17,18] and dicotyledonous [19,20,21] plants. Beans are found in all agricultural regions of Mexico, and by 2022 more than 570,000 bean producers directly participated in their cultivation. Bean growth generates more than 382,000 permanent jobs and the annual production value is around MXN 13 billion [La importancia de la producción de frijol en México|Secretaría de Agricultura y Desarrollo Rural|Gobierno|gob.mx (www.gob.mx; accessed on 1 November 2023)]. The presence of *B. bassiana* has been previously reported in bean plant field-collected samples in various Canadian locations and it may infect above- and below-ground insects throughout bean endophytic colonization [22]. Endophytic *B. bassiana* has also been shown to stimulate bean growth [23]. 

However, we have limited tools to identify the strains responsible for endophytic activity. After evaluating strains, it is common and accepted to use culture-dependent methods that do not discriminate between strains [12,13,14]. In addition, molecular techniques detect only endophyte species [24,25,26]. Therefore, it would be of great significance to use techniques that avoid a sequencing step to confirm the endophytic strain in either culture-dependent or molecular analysis.

Techniques to study natural or artificially applied endophytes include direct observation, and culture-dependent and culture-independent methods, in which plant surface microorganisms are first removed [27]. However, this process may affect the diversity [28] and viability of endophytes of interest [29] in the plant. Therefore, it is essential to use culture-independent methods for the accurate and fast detection of endophytes, even if the microorganism is not viable. 

We currently have a few methods to monitor strains after being applied as endophytes in the plant. One of these techniques utilizes the *B. bassiana* 04/01-tip strain transformed with green fluorescent protein (GFP), whose establishment in opium plants was monitored by confocal laser microscopy (CLSM) [30]. PCR and quantification by real-time qPCR were also standardized, but they did not differentiate between strains [30]. 

In another study using the sequence characterized amplified regions (SCARs) technique in combination with the culture-dependent method, detection of up to 100 pg of the non-endophytic *B. bassiana* GHA strain in soil and foliar samples was reported [31] and the *B. bassiana* ITCC4796 strain was detected from samples of the stem, leaves, and capsules of jute (*Corchorus olitorius* L.) crop. However, since it was not confirmed that the oligonucleotides were exclusive of one tested strain, it was concluded that the technique only detected *B. bassiana* [32]. Furthermore, *B. bassiana* strains established as endophytes were observed in maize plants with the amplification and sequencing of the elongation factor 1-alpha (ef1-α) gene [33]. From the techniques mentioned above, it was not always possible to distinguish between strains, and more laborious techniques were required that involved the transformation or sequencing of each strain.

Amplification of four insertion sites of group I introns, located downstream of the *B. bassiana* rDNA large subunit (LSU), has been used to determine the absence or presence of introns that may be inserted into such sites, which are called Bb1 to Bb4, and whose presence or absence profile has helped to differentiate *B. brongniartii* [34] and *B. bassiana* [35] strains in axenic cultures. 

The present study aimed to develop an in planta method to differentiate between three *B. bassiana* strains established in beans by nested PCR, using the intron insertion profile of each strain. We detected the presence of BBPTG4 and BB37 strains native to Mexico and one commercial GHA strain (Botanigard^®^ 22WP; BioWorks Inc., Victor, NY, USA). We also identified *B. bassiana* species [30], which allowed for accurate monitoring of the presence of *B. bassiana* and its strains after application, following the techniques standardized in the present study.

## 2. Results

### 2.1. Amplification of the Insertion Profile of Group I Introns in B. bassiana and Beans

**Figure 1:**
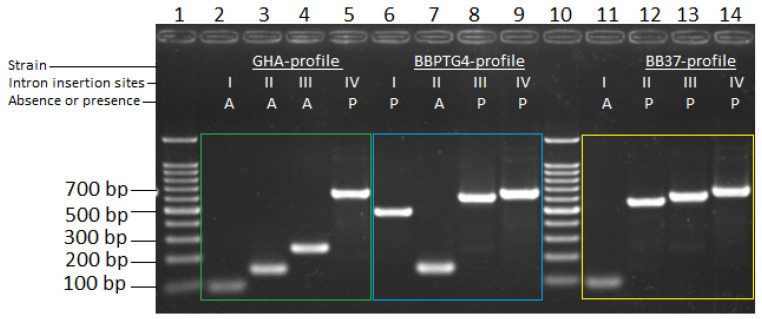
Gel electrophoresis of the amplification patterns of four group I intron-insertion sites (I–IV) located in the LSU of rDNA of three *B. bassiana* strains, with previously designed primers [35]. Lanes 1 and 10, 100 bp DNA ladder marker (Promega); lanes 2 to 5, GHA strain pattern (green square box); lanes 6 to 9, BBPTG-4 strain pattern (blue square box); and lanes 11 to 14, BB37 strain pattern (yellow square box). Intron insertion sites I–IV are marked with an “A” for absence or a “P” for the presence of introns for each strain. Amplicon sizes for the four intron insertion sites, with or without introns, are shown in Table 1.

The profile of each of the *B. bassiana* strains was determined by amplification of the four group I intron insertion sites (sites I to IV) of the 3′OH region of LSU. All strains showed different insertion profiles. When introns were present in the strain’s genome, their amplified bands were larger than 500 bp at each site (introns were named Bb1 to Bb4). However, when they were absent, they generated smaller products. Therefore, in the GHA strain, we showed the absence of introns Bb1, Bb2, and Bb3 in insertion sites I–III and the presence of the inserted intron Bb4 in insertion site IV. Furthermore, the BBPTG4 and BB37 strains showed only the absence of Bb2 and Bb1 introns in insertion sites II and I, respectively (Figure 1). 

Table 1 shows the approximate sizes of the amplified bands for each strain, according to the presence or absence of group I introns in insertion sites I to IV of the *B. bassiana* large subunit (LSU) [35]. Band sizes were the same in all strains, depending on whether the group I intron was present or not.

Amplification of the four insertion sites of group I introns was also performed in the genome of two batches of the bean *var.* Pinto Saltillo to determine if the primers were also hybridized in beans, which would be an undesirable feature. Amplifications were observed in both batches, showing strong bands for sites III and IV, and strong or faint bands for sites I and II in batches 1 and 2, respectively. Thus, the bean genome contains sequences that hybridized with the primers that amplify *B. bassiana* insertion sites and that the plant may also have insertion sites, without inserted introns, since the four bands amplified in each of the two lots (right and left red box, respectively), which amplified bands of ~84 bp, ~157 bp, ~244 bp, and ~207 bp, respectively. The results match band sizes when there are no introns I–IV inserted in *B. bassiana* (Table 1).

### 2.2. B. bassiana Strains Detection by Nested PCR

**Figure 2:**
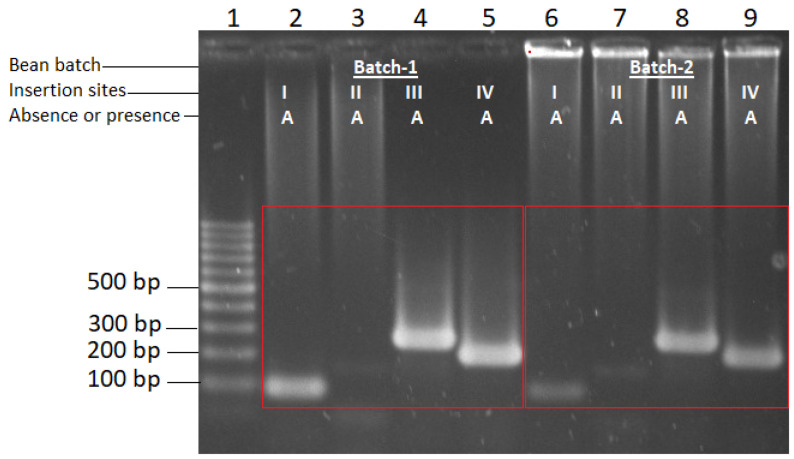
Gel electrophoresis of the amplification pattern of the four group I intron insertion sites (I–IV) of the 28S gene of *B. bassiana* in beans, with previously designed primers [35]. Pinto Saltillo from two different batches. Lane 1, 100 bp DNA Ladder marker (Promega), lanes 2 to 5 amplification of I–IV insertion sites in Pinto Saltillo beans batch-1 (red square box left; and lanes 6 to 9 amplification of insertion sites I–IV in Pinto Saltillo beans batch-2 (red square box right). Amplicon sizes for the four intron insertion sites are shown in Table 1. A, Absence of introns.

A nested PCR was designed for the detection of the intron insertion profile of *B. bassiana* as an endophyte in bean because three of the four pairs of primers to detect the intron insertion pattern of *B. bassiana* also hybridized in bean (Figure 2), which is undesirable. We also performed a test to detect *B. bassiana,* using positive controls (plant DNA mixed with *B. bassiana* DNA) by a simple PCR, which failed because the fungus DNA level was too low to be detected with a simple PCR. We used the sequence of the 3′OH region of the *B. bassiana* 28S gene (GenBank JF429894.1) for the oligonucleotide design for the first nested PCR reaction. These oligonucleotides covered the four insertion sites of this region and were hybridized from base pairs 6002 to 8134 of the sequence. 

Amplification tests for the first nested PCR reaction included Go Taq polymerase (Promega Corp., Madison, WI, USA) and the hot starts Platinum™ High Fidelity and Platinum Super Fi Mix (ThermoFisher Scientific, Carlsbad, CA, USA). The use of Go Taq polymerase was discarded because it only amplified the GHA strain, which generated a small amplicon of approximately 1550 bp. As platinum enzymes amplified the first reaction in the three strains, we continued using Platinum Super Fi in all experiments.

**Figure 3:**
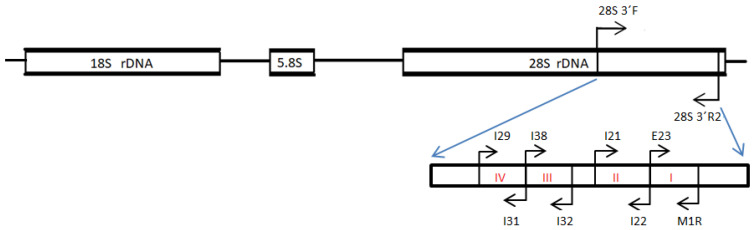
Nuclear ribosomal unit of *B. bassiana* showing the large subunit (LSU) (28S) 3′OH region, with the set of primers designed to amplify the all-region where the four group I intron insertion sites are located, and the designed primers to amplify every intron insertion site, with few modifications [35]. Intron insertion sites I–IV are shown in red. The arrows indicate the sense of primers.

The final reaction (10 µL final volume) consisted of 5 µL of 2X Platinum™ SuperFi™ PCR Master Mix (ThermoFisher Scientific), 0.5 µM of primers 28S-3′F 5′-AGC TAG GGT TTA ATG GCC GGA AGA-3′, and 28S-3′R2 5′-TAG AGG CGT TCA GCC ATT ATC CAG-3′, 2.0 µL of the 5X SuperFi™ GC enhancer, and one microliter of plant DNA with endophyte (10 to 50 ng/μL). We selected a type of touchdown PCR program that is especially used for low concentrations of the target DNA. The final program consisted of 30 s at 98 °C as the initial denaturation temperature, followed by 14 cycles of 10 s of denaturation at 98 °C, 30 s of annealing at 67 °C with a reduction of 0.7 °C for each cycle, and 2 min of extension at 72 °C, followed by 30 cycles of 10 s of denaturation at 98 °C, 30 s of annealing at 56 °C, and 2 min of extension at 72 °C, with a final extension of 10 min at 72 °C. The final product was used as a template for the second reaction, using one microliter as a sample for each of the four separate reactions used for amplification of insertion sites I–IV [35] described in Section 4.4. Positions for all amplifications for the strain-nested PCR primers in the 3′OH region of the LSU of the *B. bassiana* rDNA are shown in Figure 3.

### 2.3. Detection Limit 

**Figure 4:**
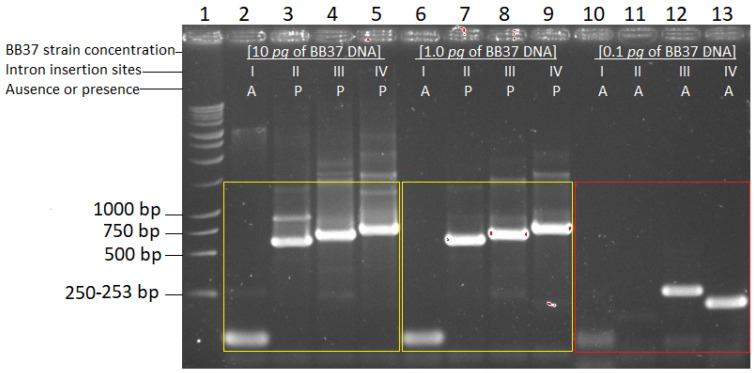
Gel electrophoresis of amplification pattern of group I intron insertion sites (I–IV) of *Beaveria bassiana* 28S gene of strain BB37 to determine the detection limit of the new strain-nested PCR technique. Lane 1, 1 kb DNA Ladder (Promega); lanes 2 to 5, 6 to 9, and 10 to 13, amplifications of four group I intron insertion sites at a concentration of 10 pg (left yellow square box), 1.0 pg (right yellow square box), or 0.1 pg (red square box) BB37, using 10 ng of bean DNA as a background for each amplification. Amplicon sizes for the four intron insertion sites, with or without introns, are shown in Table 1. A, Absence of introns; P, presence of introns.

With the conditions described above, a detection limit test was run using the BB37 strain since it had the longest first reaction amplicon and was the most difficult to amplify. In the electrophoresis gel, we observed that the detection limit was up to 1 pg of *B. bassiana* DNA for every 10 ng of bean DNA (lanes 6 to 9 in the right yellow square box, where the insertion or presence of introns in insertion sites positions II, III, and IV, and the absence in insertion site I are shown) (Figure 4). At a concentration of 0.1 pg for every 10 ng of bean DNA, it was no longer possible to observe the typical intron insertion profile for BB37 (lanes 10 to 13), since all four positions did not have inserted introns (Figure 4). 

### 2.4. Inoculation of B. bassiana in Beans

**Figure 5:**
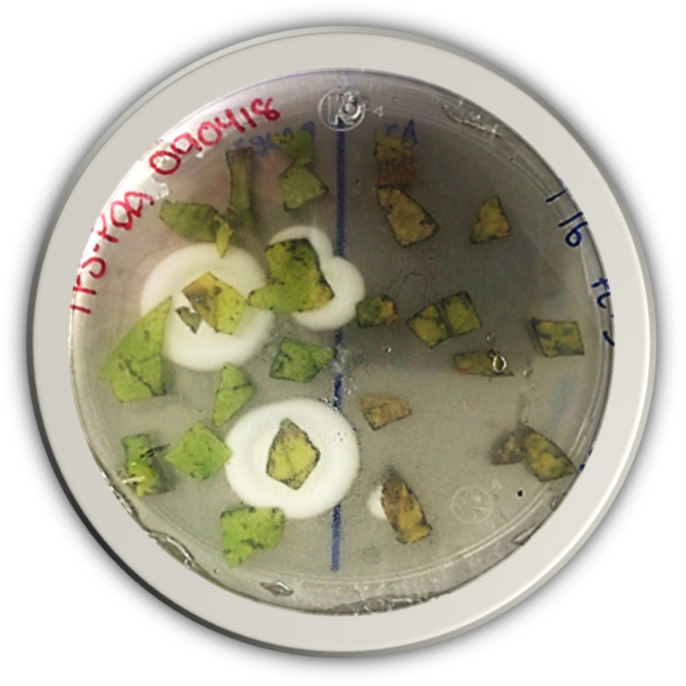
Fungal development on PDA of surface-sterilized *Beaveria bassiana*-treated bean foliar tissue cuts. Inoculated adaxial and abaxial leaf surfaces are observed on the right and left of the Petri dish.

After incubation in potato dextrose agar (PDA), we showed endophyte growth with the typical colony morphology of *B. bassiana* in some of the cultures (Figure 5).

**Figure 6:**
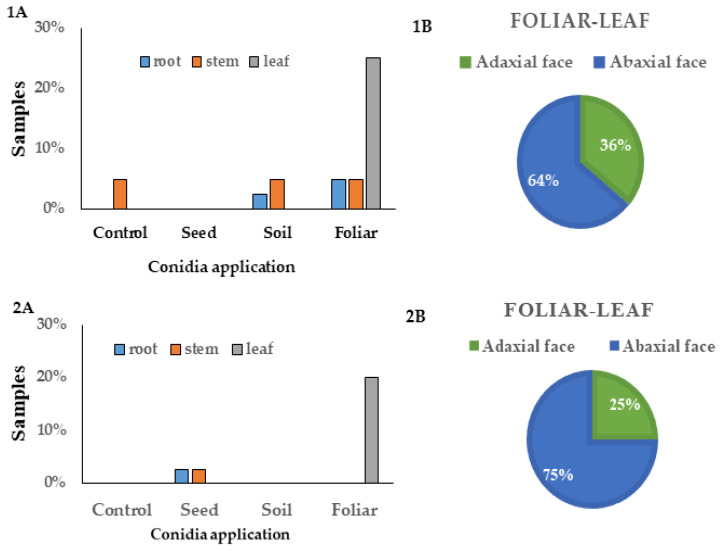
Percentage of *B. bassiana* GHA strain colonization in bean plants. Sterile soil (**1A**,**1B**) and non-sterile soil (**2A**,**2B**). (**A**) Seed, soil, and foliar (adaxial and abaxial) applications and control without inoculation. (**B**) Foliar application in adaxial and abaxial leaf surfaces is separately shown.

Tissue plating analysis showed that using the foliar spray method, 25% and 20% of GHA strain establishment in sterile and non-sterile soil, respectively, was obtained in bean plants (Figure 6(1A,2A)), and for foliar administration, a better establishment was observed when it was applied to the abaxial leaf surface (64% and 75%, respectively) than the adaxial leaf surface application (36% and 25%, respectively) (Figure 6(1B,2B)).

Regarding the application in seed and soil, we showed differences between sterile and non-sterile soil. In sterile soil, we did not observe the establishment of *B. bassiana* in any type of tissue after seed application, but it was detected in roots (2.5%) and stem (5.0%) when applied to the soil. In contrast, in non-sterile soil, we detected establishment in the roots (2.5%) and stems (2.5%) after seed inoculation, but it was not detected in any tissue when it was applied to the soil (Figure 6(1A,2A)).

### 2.5. Test of the New Nested PCR Technique with Control GHA Strain and Tissue Selection

**Figure 7:**
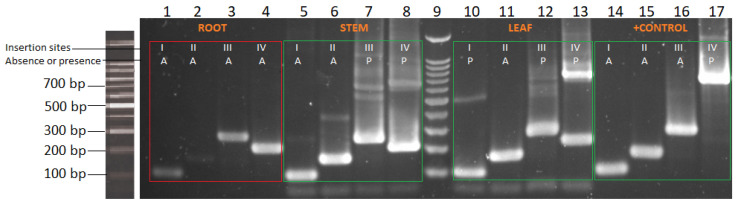
Gel electrophoresis of two-step strain-nested PCR for *Beaveria bassiana* GHA strain profile detection in non-sterile surface bean samples. Lanes 1 to 4, 5 to 8, 10 to 13, and 14 to 17 amplification bands from insertion sites I–IV, respectively, from root, stem, leaf (previously inoculated with GHA strain), and *B. bassiana* GHA strain from fungus DNA (right green square box). Lane 9, 100 bp DNA Ladder marker (Promega). Intron insertion sites I–IV are marked with an “A” for absence or a “P” for the presence of introns for each sample. Amplicon sizes for the four intron insertion sites, with or without introns, are shown in Table 1.

In order to select only one type of tissue to analyze for further tests, the intron insertion profile of the control GHA strain was determined in root, stem, and leaf samples that were previously inoculated or uninoculated to test the new strain-nested PCR technique. In a non-surface sterilized sample from a previous foliar inoculation selected for molecular analysis, a negative result was observed for the GHA strain profile in the root sample DNA, where we showed the absence of Bb1, Bb2, and Bb3 introns in insertion sites I–III and the absence of the Bb4 intron in the IV insertion site, since all positions of the insertion site bands were smaller than 500 bp (Figure 7; red square box), thus indicating that it did not have the GHA strain profile. 

In addition, some positive samples were observed, such as the profiles obtained from stem and leaf samples (Figure 7; left and middle green square boxes). In this regard, it was observed that the GHA strain was present when the Bb4 intron was inserted in the insertion IV position, showing a band of ~620 bp, as shown in the positive control (Figure 7; right green square box). However, other bands of possible introns and/or no specificities were also shown, such as the band on insertion site III of the stem and the bands in sites I and III in the case of the leaf sample. The presence of bands of more than 500 bp may indicate the existence of other endophytes or epiphytes since the sample was not surface sterilized.

As we observed that abaxial leaf surface applications were the most suitable for the establishment of the endophyte with the tissue plating technique (Figure 6) and as in the new technique, the intron insertion profile in leaf samples was well observed (Figure 7; middle green square boxes) with an intense band of the Bb4 intron in the insertion IV position, it was decided to continue with the rest of the tests only with abaxial leaf surface applications and molecular tests were only performed on leaf tissues.

### 2.6. Comparison of Techniques and Searching for Endophytic B. bassiana Strains in Bean Plants 

Information from the conventional tissue plating technique was compared with the *B. bassiana*-nested PCR technique [30] and the one we developed to differentiate among three *B. bassiana* strains, from leaf samples of nine foliar inoculated and two uninoculated (control) plants that were previously sterilized surface. It was observed that with the tissue plating technique, it was not possible to detect any positive sample that had been previously inoculated with the GHA strain. In contrast, we showed two of three and one of three BBPTG4 and BB37 strain-inoculated samples, respectively (Table 2). 

**Figure 8:**
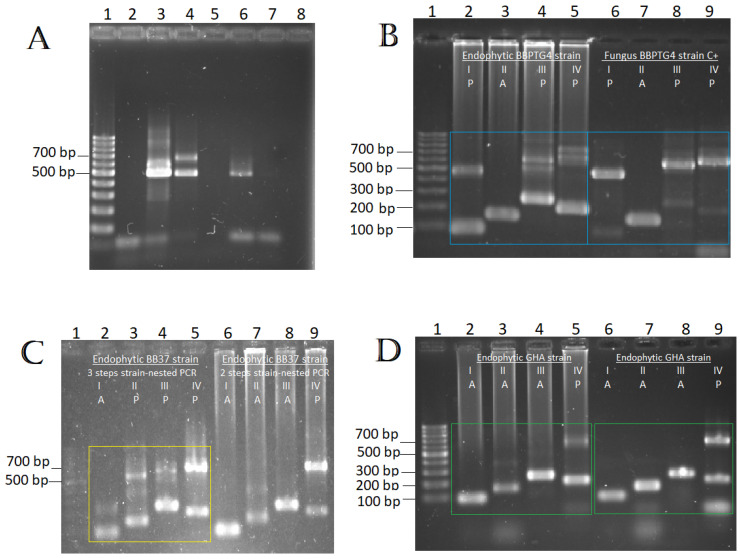
(**A**) Gel electrophoresis of Bb-nested PCR to detect samples positive for *B. bassiana* of bean plants that were previously surface-sterilized. Lane 1, 100 bp DNA Ladder marker (Promega); lanes 2 to 7, foliar samples from plants 1, 2, 7, 3, 4, and C-plant 1 (Table 2); and lane 8, negative control (without sample). Expected positive signal band of approximately 464 bp. (**B**) Gel electrophoresis of strain-nested PCR to detect the BBPTG4 profile of previously surface-sterilized leaf sample. Lane 1, 100 bp DNA ladder marker (Promega); lanes 2 to 5, three steps strains-nested PCR of sample from plant 5 (Table 2); and lanes 6 to 9, single PCR of insertion sites I–IV of DNA from fungus strain BBPTG4 (positive control). (**C**) Gel electrophoresis of three- and two-step strain-nested PCR to detect BB37 profile of a previously surface-sterilized leaf sample. Lane 1, 100 bp DNA Ladder marker (Promega); lanes 2 to 5, three-step strain-nested PCR reaction of a sample from plant 8 (Table 2); lanes 6 to 9, same sample with two-step reaction. (**D**) Gel electrophoresis of strain-nested PCR to detect the GHA profile of previously surface-sterilized leaf samples. Lane 1, 100 bp Ladder marker (Promega); lanes 2 to 5 and 6 to 9, samples from plant 4′ and 6′ respectively (Table 3), both with three steps of strain-nested PCR. To confirm all the intron insertion profiles of each strain, refer to Figure 1. Amplicon sizes for the four intron insertion sites, with or without introns, are shown in Table 1. A, Absence of introns; P, presence of introns.

For the tissue plating technique, the percentage of tissue positive for *B. bassiana* is shown in parentheses. After analyzing the bean tissue DNA of controls and treated plants with the Bb-nested PCR technique [30], we observed six positive samples, compared with the three positive samples using the tissue plating technique (Table 2), detecting the typical 464 bp band (Figure 8A) from the nested PCR selected to detect *B. bassiana* strains.

We decided to analyze samples with our technique that previously gave a positive result with the Bb-nested PCR technique [30] since the latter was more sensitive and detected *B. bassiana* species. It was observed that with the strain-nested PCR technique, two of the six positive samples detected by the *B. bassiana*-nested PCR technique [30] were obtained (Table 2).

It should be considered that extra steps were performed in the first nested PCR reaction to obtain positive results since a three-step nest PCR reaction had to be performed on these DNA samples from surface-sterilized tissues. The first reaction was performed twice, taking one microliter as a sample and further taking one microliter of the second reaction to amplify the intron insertion profile (as the third reaction). 

In the case of GHA being an endophyte, the only sample out of three that resulted positive (plant-2) with the Bb-nested PCR technique [30] gave a negative result with the strain-nested PCR technique to differentiate between strains. In the case of BBPTG-4, we detected two of three (plant-4 and plant-5) (Table 2) positive samples with the Bb-nested PCR technique [30], and it was possible to observe the characteristic intron insertion profile of the strain in one of these samples (plant-5) (Figure 8B) with the strain-nested PCR technique.

For BB37, where all the samples were positive with the Bb-nested PCR technique [30] for the detection of *B. bassiana* (plant-7 to plant-9) (Table 2), only in one sample we observed the BB37 strain intron insertion profile with the strain-nested PCR technique (plant-8). Figure 8C shows the sample from plant-8, performed with three steps (yellow square box), where it was possible to obtain the BB37 profile, and the same sample with two steps (lanes 6 to 9), where it was not possible to observe the BB37 profile. 

Furthermore, as in the previous experiment, we did not obtain positive samples with the plating method in samples previously inoculated with GHA and it was not possible to demonstrate the appearance of its intron insertion profile, with the only positive sample using the Bb-nested PCR technique [30] (Table 2). We decided to develop an experiment using only the GHA strain and testing a greater number of plants, but we did not show positive samples by the tissue plating technique (Table 3). 

Using the Bb-nested PCR technique [30], 6 out of 10 samples were obtained (samples from plants 4′ and 6′ to 10′), and of these positive samples, 4 out of 6 were observed with our method (samples from plants 4′, 6′, 9′, and 10′) (Table 3). In Figure 8D, we showed the GHA profile from surface-sterilized tissue samples. In addition, two non-inoculated controls were detected that tested positive, probably due to contamination with the treated plants [30].

## 3. Discussion 

The aim of the present study was to develop a molecular technique useful to differentiate between the *B. bassiana* strains GHA (control) and BBPTG4 and BB37 (native strains isolated from Mexican soils) *in planta*, without sequencing. Our technique was based on a nested PCR (strain-nested PCR technique), which detects the insertion pattern of four group I introns of the *B. bassiana* 28S rRNA gene, when it is as an endophyte in bean plants. Since such an insertion pattern is exclusive for every of these strains, it helps differentiate among tested strains. 

Several methods are currently available to detect *B. bassiana* strains as endophytes. One of them is transforming the EABb 04/01-Tip strain with a green fluorescent protein to monitor and observe colonized tissue by confocal microscopy [30]. Other techniques have also been standardized to detect *B. bassiana* qualitatively and quantitatively as an endophyte in opium culture by nested PCR and real-time PCR (qPCR), both of which are highly sensitive techniques (0.01 pg/10 ng of plant DNA and 26 fg/20 ng of plant DNA, respectively) [30]. The approach of the strain-nested PCR technique used in the present study was touchdown nested PCR. Our technique had a detection limit of up to 1 pg per 10 ng of plant DNA. It involves two steps for the first reaction, with 28S-3′F and 28S-3′R2 oligonucleotides, with a hot-start enzyme. For the third amplification reaction, the intron insertion profile, conditions, and oligonucleotides were adjusted from a previously reported technique [35]. The strain-nested PCR technique possessed lower sensitivity compared with the aforementioned ones, since in the amplification of the four insertion sites of group I introns we did not observed an interference with plant DNA. The strain-nested PCR technique used in this study had the advantage that it may differentiate among three different strains or possibly more with different patterns, whereas the Bb-nested PCR technique [30] only detected *B. bassiana* species. 

Another technique that detects three strains of *B. bassiana*, which are endophytic in maize plants, is based on the amplification of the elongation factor alpha-1 gene *ef1*α, using a touchdown nested PCR to detect *B. bassiana* [33]. However, this technique must use sequencing to differentiate between these strains, as well as in other studies, where ITS sequencing is common to confirm that the identity of the applied strain is that obtained as an endophyte [36,37], and one disadvantage of sequencing techniques is that they are time-consuming. Another technique detects up to 100 pg of the GHA strain per gram of soil, using oligonucleotides obtained by SCAR [31], but this technique was not used for the detection of the strain as an endophyte. The strain-nested PCR technique used in the present study was 100 times more sensitive.

Using the SCARs technique [31], the *B. bassiana* ITCC 4796 strain was detected as an endophyte in jute plants (*Corchorus capsularis* L.), but this was not confirmed to be exclusive to that strain. It was then concluded that they were useful for detecting only *B. bassiana* [32]. This is similar to another study developed in corn that also used polymorphic SCARS [31] to detect the *B. bassiana* strain Bb-13 [38]. Furthermore, a study showed that three microsatellite markers were effective in differentiating *B. bassiana* strains, but it was only tested on substrates and leaves and it was not tested as an in vivo endophyte established in the plant [39].

The amplification pattern of the four group I intron insertion sites obtained from each of the strains helped to differentiate them, since the GHA strain had only the Bb4 intron inserted. The BBPTG4 strain had the Bb1, Bb3, and Bb4 introns inserted, whereas the BB37 strain had the Bb2, Bb3, and Bb4 introns inserted. In the three tested strains, intron Bb4 was the most representative, being present in 100% of the samples, followed by intron Bb3 in 66.6% of the samples, and introns Bb1 and Bb2 being present in 33.3%. 

In a more extensive analysis of the occurrence frequency of group I introns inserted into the 28S rRNA gene in 125 *B. bassiana* strains mostly from different regions of Asia, the most frequent intron was Bb4, with an 86.4% appearance [35]. Strains with an intron insertion profile, such as that of GHA, were found with an appearance frequency of 24.8%, being in 31/125 strains (the highest frequency reported). Similarly, the BBPTG4 strain had an occurrence frequency of 8.0%, with 10/125 strains (the fifth most reported) and only 1/125 with the BB37 profile with an appearance frequency of 0.8%.

Experiments conducted with the GHA strain at *B. bassiana* establishment demonstrated that a significant rate of success was achieved when the application was administered at the foliar level, regardless of whether the soil was sterile or non-sterile. This agrees with a previous report in which *B. bassiana* was established as an endophyte in sorghum, where higher endophyte establishment was also observed by foliar applications, showing similar percentages in sterile and non-sterile soil, being slightly better in sterile soil [18]. Notably, higher success rates were observed when applications were targeted at the abaxial leaf surface as compared with those at the adaxial leaf surface. This may be due to the ease of penetration and/or the opening of the stomata in the leaves, which facilitates the entry of the microorganism into the plant, as in corn [40]. An adequate establishment of *B. bassiana* via the leaves on their abaxial leaf surface in cotton, wheat, beans, corn, tomato, and squash crops has also been reported [41]. Furthermore, studies in beans have reported better *B. bassiana* establishment after foliar application [23,42]. Therefore, we tested the strains only with foliar applications on the abaxial leaf surface of leaves and sampled leaf tissue for plating and molecular tests.

Bean plant tissues obtained from tests where the three strains were applied and in which all tissues were previously surface-sterilized, have revealed that our technique detected the intron insertion profile of each one separately, as endophytes. It was observed that the control strain (GHA) was apparently easier to detect in non-sterile surface tissue (because we observed more intense bands in gels and just a two-step strain-nested PCR was required) than in surface-sterilized tissue, since a three-step strain-nested PCR was needed. However, in non-surface-sterilized samples, other possible intron bands or inspecificities from other endophytes or epiphytes may make it difficult to detect the intron insertion profile. However, using the surface-sterilized tissue and the three-step strain-nested PCR, we achieved a clean and easy way to detect the control strain GHA profile. It was considered that our monosporic GHA, even with adequate viability, may have suffered some genetic wear that did not allow it to establish itself in a Petri dish only in the last two tests. However, it was possible to detect it molecularly.

The adaptation of the three-step strain-nested PCR technique, used in this study, from samples that were previously positive with the Bb-nested PCR technique [30], was useful since the latter technique was more sensitive and detected *B. bassiana* species. One of the disadvantages of our technique is that not all samples that test positive were detected with the Bb-nested PCR technique [30] because the strain-nested PCR technique was less sensitive. However, this technique may be coupled with those already used to provide greater certainty that the applied strain is the one established as an endophyte, without requiring molecular sequencing.

Our strain-nested PCR technique also complemented the traditional technique of plating tissue, since the frequent use of sodium hypochlorite to sterilize tissue surfaces may affect the diversity of fungal endophytes [28] or result in non-viable applied endophytes, as observed in our results. Subsequent evaluations will be essential to authenticate the efficacy of this method in the field-based monitoring of single or multiple strains in beans or other plants. 

## 4. Materials and Methods

### 4.1. Fungal Isolates

*Beauveria bassiana* isolates used in this study were a commercial Botanigard^®^Bb-GHA product, a native strain from Nuevo León Mexico (BBPTG4), and a strain provided by the State Committee for Plant Health of the state of Guanajuato, Mexico (CESAVEG) called BB37. Monosporic cultures of each strain were separately prepared and preserved in sterile mineral oil at 4 °C [43]. 

### 4.2. Plant Material

Bean *(P. vulgaris*) var. Pinto Saltillo seeds were provided by the Academy of Production and Use of Crops of the School of Agronomy at the Autonomous University of Nuevo Leon, Mexico. They were disinfected with 0.5% sodium hypochlorite solution (CloralexTM, Industrial Alen, Santa Catarina, Nuevo León, México for two minutes, 70% ethanol for two minutes, rinsed three times with distilled water, and dried before testing. The last rinse was incubated in potato dextrose agar (PDA; BD Difco, México) for 10 d to determine the effectiveness of sanitization of the seed surfaces. Only surface microorganism-free seeds were used.

### 4.3. Fungi and Plant DNA Extraction and Quantification

For *B. bassiana* strain DNA extraction, a batch of monosporic cultures was grown in 100 mL of potato dextrose broth (PDB; BD Difco) for 10 d, after which DNA was extracted using the Wizard DNA purification kit (Promega Corp., Madison, WI, USA). The protocol used was the one described for the extraction of DNA from plants, with a slight adaptation of the technique in the initial part, by macerating the microorganism pellet with a lysis buffer and a pestle for two minutes, and continuing the technique as described in the regular protocol.

Plant tissue DNA was obtained from the root, stem, or leaves, depending on the experiment as follows: plant tissue was previously rinsed and placed in a 2.0 mL microtube, after which one milliliter of 2% cetyltrimethylammonium bromide (CTAB) lysis buffer (Promega Corp.), previously heated to 65 °C, was added, macerated with a pestle for five minutes, and incubated for five minutes at room temperature. Next, it was incubated on ice for five minutes, and 20 μL of RNase A (Promega Corp.) was added, mixed, and incubated at 37 °C for 20 min. We then added 3 µL of proteinase K (Promega Corp.) and mixed and incubated it at 65 °C for 30 min, after which we incubated it on ice for five minutes, followed by the addition of 600 µL of phenol-chloroform-isoamyl (25:24:1), mixing by inversion, and centrifuging at 14,000 rpm/12 min at 8 °C. We then carefully recovered 200 µL to 400 µL of supernatant, transferred it to a new 1.5 mL tube, added 50 µL to 100 µL of 10 M sodium acetate, and mixed by inversion. Next, 500 µL to 1000 µL of 100% isopropanol were added and mixed by inversion, after which the samples were stored for two hours at −20 °C for DNA precipitation. After incubation, samples were centrifuged at 14,000 rpm/15 min at 8 °C and decanted, followed by the addition of 1 mL of previously cooled 70% ethanol, incubating for five minutes at room temperature, centrifuging at 14,000 rpm/5 min at 8 °C, and decanting, after which samples were allowed to dry. Pellets were then hydrated with 30 µL to 50 µL of nuclease-free water. For fungi and plant DNA, the quality and quantity of DNA were measured by spectrophotometry in a Nanodrop Lite (ThermoFisher Scientific, Waltham, MA, USA), following the supplier’s instructions.

### 4.4. Amplification of Group I Intron Insertion Sites of LSU of B. bassiana

To determine the insertion pattern of four group I introns of the 3′OH region of the 28S rDNA of the three strains and to test if primers were amplified in the bean plant genome, four separate reactions were performed on each sample to amplify the LSU insertion sites I–IV of *B. bassiana*, as previously reported [34], using the oligonucleotides E23F 5′-CCG AAG GAA TTC GGT AAG CG-3′, and M1R 5′-GGT AAA ACT AAC CTG TCT CAC G-3′ to amplify intron insertion I, 121F 5′-CGA TCC TTT AGT CCC TCG AC-3′, and 122R 5′-CGC TTA CCG AAT TCC TTC GG-3′ to amplify insertion site II, 138F 5′-ATG GGC TTG GCA GAA TCA GCG-3′ and 132R 5′-CAG CCA AAC TCC CCC CTG-3′ to amplify insertion site III, and 129F 5′-CTG CCC AGT GCT CTG AAT GTC-3′ and 131R 5′-CGC TGA TTC TGC CAA GCC CAT-3′ to amplify insertion site IV. Each PCR reaction (20 µL final volume) contained 10–20 ng genomic DNA, 0.5 µM of each primer, and 10 µL of 2X Go Taq Green Master Mix (Promega Corp.). 

The cycling parameters were programmed in a T100 Thermal Cycler (BioRad, Hercules, CA, USA) as follows: an initial denaturation for 4 min at 95 °C, 35 cycles of 45 s of denaturation at 94 °C, 45 s of annealing at 57 °C, and 45 s of extension at 72 °C, followed by a final extension for 4 min at 72 °C. The resulting reactions were run on a 1% agarose gel at 119 V/60 min with ethidium bromide staining for at least 20 min and visualized on a BioRad Gel Doc™ XR+ Gel Documentation System photo documenter (BioRad). 

### 4.5. Design of B. bassiana Primers for the First Nested PCR Reaction and Running Programs Test

For the first nested PCR reaction, oligonucleotides were designed that encompassed the four intron insertion sites of the 3′OH of the LSU region of the rDNA of *B. bassiana* that do not hybridize in the bean genome. For the design of the primers, the Amplifix 1.5.4 program and sequences of the LSU of *B. bassiana* rDNA previously reported were used. We confirmed that selected oligonucleotides did not hybridize with the bean genome by the NCBI BLAST tool, and different running programs were tested. 

### 4.6. Sensitivity of the Nested PCR Technique 

To determine the sensitivity of nested PCR, we prepared positive controls containing 10 µg of bean DNA as a background, and 0.1 pg, 1 pg, and 10 pg of *B. bassiana* from the BB37 strain. Previously standardized strain-nested PCR was used to determine the concentration of the Bb37 strain intron insertion pattern that was no longer visible in the electrophoresis gel. 

### 4.7. Inoculation of GHA Control Strain in Bean Plants 

To determine the most effective inoculation technique to establish *B. bassiana* as an endophyte in bean plants var. Pinto Saltillo, under sterile and non-sterile soil conditions, stocks of the GHA control strain were prepared at a concentration of 1 × 10^8^ spores/mL and >80% viability, using 0.02% Triton X100 to inoculate seeds, soil, and leaves. For these, bean plants were grown in 1-L pots with a mixture of 80% soil and 20% sand. Next, three plants were planted per pot per experiment, but only one randomly selected was tested. That is, the test was carried out only once, with some replicates of different plants (but in the same experiment). Previously surface sterilized seed was planted per pot (four pots per treatment) and placed under a Philips Day-Brite T5 54W/6500 fluorescent lamp (9200 lux approximate illumination) at a height of 70 cm in a 12 h:12 h light-dark cycle. Plants were irrigated with 100 mL of water every two days and fertilized with a 6 g/L NPK 15-15-15 solution on days 10 and 22 after germination.

For seed inoculation, seeds were immersed in 10 mL of spore stock for five minutes and allowed to dry before planting. Soil and foliar treatments were performed 21 d and 32 d after germination, applying 10 mL of spore stock on the stem base and 10 mL sprayed on the leaves. In the case of foliar applications, they were tested on the adaxial and abaxial leaf surfaces of the second and third true leaves, respectively, on the same plant. Following 10 d of the last soil or foliar application, the experiment was completed, and the root, stem, and leaves were sampled, surface sterilized, and plated on PDA for further analysis.

### 4.8. Detection of Endophytic B. bassiana by Platting Tissue

We used a previously reported method [42], with some modifications, to sterilize the surfaces of roots, stems, and leaves. For this, tissues were sampled cutting pieces of 3 to 4 cm and rinsed under running water for one to two minutes to mainly remove dirt, after which further washings were performed under sterile conditions, using the following protocol for each tissue: tissues were vortexed in 20 mL of 0.5% sodium hypochlorite solution (Industrial Allen) for 1 min. They were then manually shaken in 20 mL of 70% ethanol for 2 min and three consecutive washes with 30 mL of sterile distilled water for 10 to 15 s each. After washing, some samples were stored at −80 °C for molecular analysis. To determine the effectiveness of the washing technique, 200 µL of the last rinse was incubated in PDA without antibiotics for 10 d, which must not show microbial growth. 

After surface sterilization, bean samples of root, stem, and leaf tissues were cut in approximately 0.5 cm × 0.5 cm sections and incubated PDA with 2 mg/L tetracycline, penicillin, and streptomycin solution (Sigma-Aldrich, St. Louis, MO, USA) for 15 d to determine the percentage of *B. bassiana* colonization in the host plant as an endophyte, through observation of morphology of the colonies and under the microscope. For the colonization percentage, we used the following formula:

% colonization = number of positive tissue pieces with *B. bassiana* × 100/ number of total pieces of platted tissue

### 4.9. Test of the New Nested PCR in Planta with Control Strain GHA 

To test the new nested PCR technique in the detection of *B. bassiana in planta*, extracted DNA from uninoculated and Bb-GHA-inoculated plant, tissues were processed by nested PCR, using in the first reaction the designed oligonucleotide and the selected running program, and in the second reaction, the four pairs of primers for the group I intron insertion sites [35] to detect the insertion profile of the GHA strain. This test was performed on non-surface-sterilized tissues. We then selected only one type of tissue to be sampled and analyzed for the rest of the experiments.

### 4.10. Endophytic Detection of B. bassiana Strains with New Strain-Nested PCR in Planta

To test the new nested PCR technique with the three strains, beans plants were grown as described in Section 4.7, with one plant per pot, and the strains GHA, BBPTG4, and BB37 were inoculated separately on the abaxial leaf surfaces. Experiments were performed in triplicate with two control pods. Leaves were collected and washed, as described above, and subjected to plating (Section 4.8), *B. bassiana* detection-nested PCR [30], and standardized nested PCR to differentiate among such strains. Our technique was performed only in previously positive Bb samples [30]. We also performed an extra experiment using only the GHA strain, and up to 10 bean plants were planted in separate pots and followed the same procedure described in this section.

### 4.11. Endophytic Detection of B. bassiana-Nested PCR in Planta

We performed nested PCR to detect *B. bassiana* species [30] with slight modifications. We used the universal oligonucleotides ITS1F and ITS4 for the first reaction. The reaction solution (15 µL) contained 20 ng to 50 ng of genomic DNA, 0.5 µM of each primer, and 7.5 µL of 2X Go Taq Green Master Mix (Promega Corp.), following an initial denaturation for three minutes at 95 °C, 40 cycles of denaturation for one minute at 94 °C, annealing for one minute at 61 °C, and extension for one minute at 72 °C, followed by a final extension for five minutes at 72 °C. For the second reaction, we used the oligonucleotides BBfw and BBrv, which are specific for *B. bassiana* and were designed to be nested in the ITS1F/ITS4 reaction, using the same volumes and reagents of the previous reaction and one microliter of the first reaction as the sample. Cycling parameters included an initial denaturation for two minutes at 95 °C, 40 cycles of denaturation for one minute at 94 °C, annealing for one minute at 65 °C, and extension for one minute at 72 °C, followed by a final extension for five minutes at 72 °C. The second reaction was run on a 1% agarose gel at 119 V/60 min with ethidium bromide staining for at least 20 min and visualized on a Biorad Gel Doc™ XR+ Gel Documentation System photo documenter (BioRad). 

## 5. Conclusions

The three-step strain-nested PCR technique used in this study was useful for detecting and differentiating three strains as bean endophytes that have a different group I intron insertion profile and complement the tissue plating and the Bb-nested PCR techniques [30]. To rely on the results after using this technique, it is very important to sterilize the tissue surface to remove epiphytes that may cause interference. We are confident that our results showed the applied strain and not the naturally occurring endophyte strains. Further studies should be developed to determine whether this tool is helpful for strain field monitoring in beans or other crops.

## Figures and Tables

**Table 1:** Approximate sizes of amplified bands due to the absence or presence of group I introns of *B. bassiana* LSU.

	LSU Intron Insertion Sites			
	I	II	III	IV
Intron absence	84 pb	157 pb	244 pb	207 pb
Intron presence ^1^	501 pb	656 pb	606 pb	620 pb

^1^ Amplified bands greater than 500 bp showed insertion of an intron in sites I–IV.

**Table 2:** Culture test and molecular detection of *Beauveria bassiana* and its strains from previously inoculated bean plants.

Foliar Samples	Platting Tissue	Bb-Nested PCR *	Strain-Nested PCR **
C-plant 1	Negative	Negative	NT
C-plant 2	Negative	Negative	NT
Plant-1	Negative	Negative	NT
Plant-2	Negative	Positive	Negative
Plant-3	Negative	Negative	NT
Plant-4	Positive (33%)	Positive	Negative
Plant-5	Positive (28.5%)	Positive	Positive
Plant-6	Negative	Negative	NT
Plant-7	Negative	Positive	Negative
Plant-8	Positive (11%)	Positive	Positive
Plant-9	Negative	Positive	Negative

C-plants = Control plants 1 and 2 without inoculation. Plants: GHA-treated = plants 1–3, BBPTG4-treated = plants 4–6, and BB37-treated = plants 7–9. The percentage of positive growth of endophytic *B. bassiana* in the platting tissue technique is provided in parentheses. NT = Not tested. * Bb-nested PCR = nested PCR to detect *B. bassiana* [30]. ** Strain-nested PCR = our nested PCR to detect strains GHA, BBPTG4, and BB37.

**Table 3:** Culture growth and molecular detection (nested PCR) of *Beauveria bassiana* or GHA strains from inoculated bean plants.

Foliar Samples	Plating Tissue	Bb-Nested PCR *	Strain-Nested PCR **
C-plant 1′	Negative	Positive	Negative
C-plant 2′	Negative	Positive	Negative
C-plant 3′	Negative	Negative	NT
C-plant 4′	Negative	Negative	NT
Plant-1′	Negative	Negative	NT
Plant-2′	Negative	Negative	NT
Plant-3′	Negative	Negative	NT
Plant-4′	Negative	Positive	Positive
Plant-5′	Negative	Negative	NT
Plant-6′	Negative	Positive	Positive
Plant-7′	Negative	Positive	Negative
Plant-8′	Negative	Positive	Negative
Plant-9′	Negative	Positive	Positive
Plant-10′	Negative	Positive	Positive

C-plants’ = Control plants 1′ to 4′ without inoculation, plants 1′–10′ previously GHA treated. NT = Sample non-tested. * B. bassiana-nested PCR = nested PCR to detect *B. bassiana* [30]. ** Strain-nested PCR = our nested PCR to detect strains of GHA.

## Data Availability

Data are contained within the article.
